# Comparison of proximal femoral nail antirotation and total hip arthroplasty in the treatment of femoral intertrochanteric fracture

**DOI:** 10.12669/pjms.38.4.5830

**Published:** 2022

**Authors:** Xuejun Li, Jian Xu

**Affiliations:** 1 Xuejun Li, Department of Orthopedics, Fuyang People’s Hospital, Fuyang 421002, Anhui Province, P.R. China; 2 Jian Xu, Department of Orthopedics, Fuyang People’s Hospital, Fuyang 421002, Anhui Province, P.R. China

**Keywords:** Intertrochanteric fracture of femur, Proximal femoral nail antirotation, Total hip arthroplasty

## Abstract

**Objectives:**

To investigate the effects of proximal femoral nail antirotation (PFNA) and total hip arthroplasty for the treatment of femoral intertrochanteric fractures.

**Methods:**

Clinical data from 110 femoral intertrochanteric fracture patients treated at our hospital between January 2019 and July 2020 were analyzed retrospectively. Patients were divided into two groups based on the type of surgical intervention used. One group included patients (n= 59) who had undergone PFNA internal fixation and another group (n=51) included patients who had undergone total hip arthroplasty. Perioperative situation, joint function progression, and complication incidence were assessed.

**Results:**

Total hip arthroplasty group was associated with longer operation durations, longer incisions, and more intraoperative blood loss than PFNA group (P<0.05). Joint function and pain scores in the total hip arthroplasty group were superior than PFNA group (P<0.05). The Harris score of total hip arthroplasty group was significantly higher than that of PFNA group at three, six and 12 months after operation (P<0.05). The rate of complications in patients after total hip arthroplasty was lower than that of PFNA group (P<0.05) within 12 months of the surgery.

**Conclusion:**

PFNA and total hip arthroplasty can both achieve good results for treatment of femoral intertrochanteric fractures. PFNA offers less trauma and shorter operations, while total hip arthroplasty offers advantages in terms of more rapid limb function improvements and shorter rehabilitation processes. The two kinds of surgery have advantages, and the clinical needs to have a careful look at various factors and choose the appropriate operation method.

## INTRODUCTION

With an aging population, femoral intertrochanteric fractures are becoming more prevalent, posing a major threat to quality of life.^1^,^2^ As conservative treatment is associated with the risk of complications such as pulmonary infection, bedsores, urinary calculi and lower extremity venous thrombosis, surgical treatment is recommended.^3^,^4^ Proximal femoral nail antirotation (PFNA) and total hip arthroplasty are both important surgical schemes for the clinical treatment of femoral intertrochanteric fractures. PFNA fixation has the advantages of stability, compression resistance, anti-rotation and limited trauma. However, intramedullary fixation is associated with many complications, such as bone nonunion and internal fixation failure, which are limiting its clinical application.^5^,^6^ Moreover, patients with unstable fracture and severe osteoporosis benefit less from the procedure, along with increased risk of complications.^7^ With the progress of modern tissue biology technologies, total hip arthroplasty technique became widely used to surgically treat femoral intertrochanteric fractures. It uses bone cement to fix normal bone instead of the diseased joint. Although this helps to improve hip function, the technique creates more trauma than PFNA.^8^,^9^

The study aimed to better understand the clinical value of PFNA and total hip arthroplasty for femoral intertrochanteric fracture treatment, this study retrospectively examined and summarized clinical data from patients with femoral intertrochanteric fracture.

## METHODS

The records of 110 patients with femoral intertrochanteric fracture treated in our hospital from January 2019 to July 2020 were selected, including 60 males and 50 females. Age of the patients ranged from 58 to 76 years, with an average age (65.69±4.06) years. This study was approved by the ethics committee of our hospital (Approval number: 2021125, Date: 2021-07-20).

### Inclusion criteria:

- Patients with surgical indications must have been treated using surgery, complete clinical data profiles must have been available,
- Patients must have no skin defects at the operation site, show a clear cause of injury, and had a unilateral fracture rated on the Evans classification scale as a Type II-IV.^10^

### Exclusion criteria:

- Patients with other fractures, open fractures, pathological fractures, myelopathy, coagulation dysfunction, bone tumors, osteomyelitis, bleeding tendency, and vascular injury.

### Proximal femoral nail Antirotation:

Patients were operated in supine position and were given general anesthesia. The ipsilateral hip was internally rotated to 15°, and the intertrochanteric fracture was reset under C-arm fluoroscopy guidance. A longitudinal incision 3~5cm long was made from the top of the greater trochanter toward the proximal side after satisfactory reduction. A rhombus-shaped awl was used to drill a hole at the front and middle 1/3 between the tip of the greater trochanter and the sinus piriformis. X-ray machine confirmed the position of the guide needle and expanded the medullary cavity. Then insert the proximal femoral nail, which was matched with the femoral bone marrow cavity. The nail end was placed parallel to the tip of the greater trochanter. The femoral neck screw and hip screw guide needle were inserted under X-ray fluoroscopy, and the guide needle was located approximately 5mm below the femoral head. After ensuring accurate lateral and anteroposterior positioning, the proximal helical blades and a distal locking screw were inserted, and the incision was closed layer by layer. After this, the wound was washed, bleeding staunched, and incision closed.^11^ The PFNA material was provided by the Angel Company and Trauson Company (Jiangsu, China)

### Total hip arthroplasty:

Patients underwent nerve block or spinal anesthesia in the supine position with the contralateral healthy hip fixed to maintain positioning. A lateral hip approach was implemented, layer-by-layer incisions were made to expose the fracture site, the joint capsule was cut, femoral neck osteotomy was performed, and the femoral trochanter fractures were reduced and fixed with cerclage wire. Femoral medullary cavity was expanded, false mold tested, the fracture repaired, the tightness of the prosthesis tested as well as the length of the limb, whether the prosthesis is dislocated and other indicators. After the bone marrow cavity was thoroughly washed, it was filled with bone cement, the rake angle was maintained, joint prosthesis installed, stability tested, timely cleanup of the excess cement monitored. After solidification, the stability of the prosthesis was reevaluated. Tightness of the joint, length of both lower limbs, etc were checked for displaced greater trochanter fractures. Steel wires or non-absorbable wires were used for reconstruction and fixation. The bleeding was then staunched, and the incision was washed and closed.^12^ The biotype artificial joint was provided by the Zhengtian Company (Tianjing China) and the Link Company (Germany).

### Postoperative Treatment:

Antibiotics were given to prevent infection. Low molecular weight heparin sodium was given to prevent venous thrombosis. Patients were instructed to exercise quadriceps femoris and ankle function.

The general clinical data and postoperative related indexes were collected, including: 1) Perioperative conditions, including operation time, intraoperative blood loss, incision length, out of bed time, weight-bearing exercise time and hospital stay; 2)The hip function scores of the two groups at three, six and 12 months after operation. The hip function of the patients was evaluated by Harris scale, with a higher score indicating better recovery of the hip function, and a total score of 100 points;^13^ 3)The incidence of complications within 12 months after operation .

Statistical Analysis was done using SPSS v.22.0 software. Measurement data were expressed as (x̄±s), with t-test used to examine statistical differences. Count data were expressed as n(%) and evaluated using the *χ^2^* test. P<0.05 indicates a statistically significant difference.

## RESULTS

The two patient groups, divided on the basis of surgical protocol, did not differ in terms of gender distribution, age, Evans classification, body mass index (BMI), or other clinical metrics (P>0.05, Table-I). Total hip arthroplasty group was associated with longer operation times, more intraoperative blood loss, and longer incision lengths than PFNA group (P<0.05). However, patients in the total hip arthroplasty group were able to meet recovery metrics, including out-of-bed time, weight-bearing exercise time, and hospital stay duration, faster than patients in the PFNA group (P<0.05, Table-II). Moreover, the Harris score of total hip arthroplasty group was significantly higher than that of PFNA group at three, six and 12 months after operation (P<0.05, Table-III). Finally, total hip arthroplasty the complication rate of total hip arthroplasty within 12 months was lower than that of PFNA group (5.08% vs 17.95%, P<0.05, Table-IV).

**Table-I:** Comparison of general clinical data between the two groups[n(%),x¯±s].

Group	n	Sex (M/F)	Age (years)	Evans type			Cause of injury			BMI (kg/m^2^)

				II	III	IV	Heavy injury	Fall from height	Traffic accident	
Total hip arthroplasty group	59	35/24	65.84±3.88	16(27.12)	30(50.85)	13(22.03)	19(32.20)	15(25.42)	25(42.37)	24.18±1.93
PFNA group	51	31/20	65.51±4.30	19(37.25)	22(43.14)	10(19.61)	12(23.53)	13(25.49)	26(50.98)	23.84±1.98
t		0.024	0.433		1.304			1.167		0.899
P		0.876	0.666		0.521			0.558		0.371

**Table-II:** Comparison of perioperative conditions between the two groups (x¯±s).

Group	n	Operation time (min)	Intraoperative blood loss (ml)	Incision length (cm)	Out of bed time (d)	Weight bearing exercise time (d)	Length of stay (d)
Total hip arthroplasty group	59	72.15±8.22	224.95±32.85	10.49±1.60	10.45±2.05	12.14±1.76	14.57±1.92
PFNA group	51	56.12±7.89	140.61±19.26	4.47±1.69	17.71±2.75	22.06±3.12	20.78±3.08
t		10.394	16.681	19.169	15.883	20.122	12.446
P		<0.001	<0.001	<0.001	<0.001	<0.001	<0.001

**Table-III:** Comparison of Harris score between the two groups (x¯±s, score).

Group	n	three months after operation	six months after operation	12 months after operation
Total hip arthroplasty group	59	68.51±5.49	79.27±6.01	84.95±5.99
PFNA group	51	54.78±4.74	60.67±5.25	65.31±5.64
t		13.926	17.149	17.613
P		<0.001	<0.001	<0.001

**Table-IV:** Comparison of the incidence of complications between the two groups within 12 months after operation [n (%)].

Group	n	Subcutaneous edema	Pressure sore	Infected	DVT	Total incidence
Total hip arthroplasty group	59	1(1.69)	0(0.00)	2(3.39)	0(0.00)	3(5.08)
PFNA group	51	3(5.88)	1(1.96)	4(7.84)	1(1.96)	9(17.95)
χ^2^						4.442
P						0.035

## DISCUSSION

This study found that PFNA internal fixation and total hip arthroplasty have certain advantages in the treatment of femoral intertrochanteric fractures. PFNA was associated with improved operation duration and less intraoperative blood loss than total hip arthroplasty. However, total hip arthroplasty was more effective in terms of time taken to regain ambulatory status, time taken to weight-bearing exercise, total hospital stay duration, and joint function.

PFNA has significant advantages when it comes to reducing surgical trauma. Accordingly, PFNA resulted in less intraoperative blood loss than total hip arthroplasty. Shin Yoon Kim et al.^14^ compared the results of long-stem cementless calcar-replacement hemiarthroplasty with those of treatment with a PFNA for unstable intertrochanteric fractures in elderly patients. Their results showed that patients with PFNA had shorter operation time, less bleeding and fewer blood transfusion units. PFNA is a widely used intramedullary fixation system. The main nail of PFNA has a 6°valgus angle design, making it easy to insert into the femoral medullary cavity. After driving the main nail, only a spiral blade needs to be driven into the femoral neck and a locking nail needs to be screwed into the distal end. The operation is simple and the clinical effect is remarkable.^15^ However, PFNA has some shortcomings like other internal fixation methods. If the preoperative reduction is poor, the fracture site is easily separated when the spiral blade is driven in, and the length and insertion depth of the spiral blade should be appropriate to avoid screwing in the spiral blade too deep and thus difficult to pull out. For patients with severe comminuted fractures, PFNA is less effective in achieving ideal fixation effect, fracture stability is poor, postoperative fracture healing time is long, and the sheer force of steel plate is large, which increases the risk of internal fixation nail penetrating bone and cutting bone to a certain extent.^16^,^17^

In recent years, total hip arthroplasty has been widely used in the treatment of unstable femoral intertrochanteric fractures in the elderly.^18^ During the procedure of total hip arthroplasty, the bone cement can solidify rapidly, achieve the purpose of immediate stability. This allows patients to get out of bed early and carry out functional exercise, and ensures that patients can perform weight-bearing exercise as soon as possible. Although the operation is traumatic, it can reduce the load area of the prosthesis, help to reconstruct the femoral calcar and effectively stabilize the fracture block in a short time. The integrity of the bone cortex of the proximal femur is restored, and the proximal support effect issatisfactory.^19^,^20^ Keating JF et al.^21^ conducted a multicenter clinical trial of 298 patients with hip displaced subchondral fractures. At four and 12 months after the operation, patients undergoing total hip arthroplasty performed significantly better than those treated with intramedullary nail fixation in joint function recovery and health status. In addition, Cao L et al.^22^ found that total hip arthroplasty can reduce the incidence of complications and reoperation and better restore hip function in a trial of 285 patients with hip fractures over the age of 65.^23^ Therefore, the corresponding surgical methods should be selected according to the clinical conditions of patients, so as to ensure the effectiveness and safety of treatment and improve the effect of limb function.

### Limitations:

We did not clarify the difference in efficacy of the above two surgical schemes in patients of different ages. Furthermore, the study follow-up time was only one year, and was retrospective in nature. Therefore, additional prospective studies with larger sample sizes are required to confirm our observations.

## CONCLUSION

This study found that intramedullary nail fixation and hip arthroplasty can both achieve good results for treating femoral intertrochanteric fractures. However, while the trauma induced during intramedullary nail fixation is less than that of hip arthroplasty, hip arthroplasty has advantages in improving limb function and shortening the rehabilitation process with lower rate of complications. These results may provide a reference for relevant clinical practitioners in their decision-making processes.

### Authors’ Contributions:

**XL:** Conceived and designed the study.

**XL & JX:** Collected the data and performed the analysis.

**XL:** Was involved in the writing of the manuscript and is responsible for the integrity of the study.

All authors have read and approved the final manuscript.
